# Mesoporous Bioactive Glass Nanoparticles in the SiO_2_-P_2_O_5_-CaO-MO (M=Mg, Zn) System: Synthesis and Properties

**DOI:** 10.3390/jfb13040180

**Published:** 2022-10-07

**Authors:** Andrada-Ioana Damian-Buda, Cristina-Daniela Ghițulică, Andreia Cucuruz, Georgeta Voicu, Daniela Culita, Victor Fruth-Oprișan, Lucian Toma Ciocan

**Affiliations:** 1 Department of Biomaterials and Medical Devices, Faculty of Medical Engineering, Politehnica University of Bucharest, 1-7 Gh. Polizu Street, RO-060042 Bucharest, Romania; 2 Department of Science and Engineering of Oxide Materials and Nanomaterials, Faculty of Chemistry Engineering and Biotechnologies, Politehnica University of Bucharest, 1-7 Gh. Polizu Street, RO-060042 Bucharest, Romania; 3 Institute of Physical Chemistry “Ilie Murgulescu”, Splaiul Independenței, no.202, RO-060021 Bucharest, Romania; 4 Department of Prosthetics Technology and Dental Materials, Carol Davila University of Medicine and Pharmacy, 8 Eroii Sanitari Street, RO-050474 Bucharest, Romania

**Keywords:** nanoparticles, mesoporous bioactive glass, antibacterial activity

## Abstract

Mesoporous bioactive glass nanoparticles (MBGNs) are widely recognized for their ability to bond to hard tissue, while the ions released from the BG structure enhance specific cellular pathways. In this study, the SiO_2_-P_2_O_5_-CaO-MgO-ZnO system was used to successfully synthesize MBGNs by a microemulsion-assisted sol-gel method. The MBGNs calcinated at 600 °C/3 h had a typical phosphosilicate structure together with a poorly crystalline hydroxyapatite (HAp). The addition of ZnO not only led to a higher degree of crystallinity of HAp but also induced a higher porosity of the particles. All MBGNs had a mesoporous structure with an interconnected network of slit shape pores. For each type of composition, two families of highly dispersed spherical nanoparticles could be identified. In vitro tests in simulated body fluid (SBF) proved that after only 3 days of immersion all the materials were covered with a layer of brushite whose degree of crystallinity decreases in the presence of Zn^2+^. The antibacterial assay revealed a strong inhibitory effect for all samples after 40 h of contact. Simultaneously, MBGNs did not increase the intracellular oxidative stress while it stimulated the cell proliferation process.

## 1. Introduction

In 2050, the number of women who will be suffering from osteoporosis is expected to rise by 240%, with a sharper increase of over 310% for men [1]. Even though it is known that hard tissue can self-regenerate, when the bone defect is larger than 25 mm, the cells lose their ability to substitute the whole amount of lost tissue [2]. Thus, synthetic bone grafts are currently used. Among different biomaterials approved for bone substitution, bioactive glass (BG) stands out as the first material that was able to create a strong interfacial bond with the bone [3,4].

Since its discovery 50 years ago, incredible progress has been made not only in understanding the underlaying biological mechanism of BG activity but also in the development of complex systems that could assure the control release of drugs [5,6,7]. One such multifunctional platform is represented by mesoporous bioactive glass nanoparticles (MBGNs), which are characterized by remarkable high surface area and pore volume [8,9]. Because of these unique textural proprieties, the bioactive response is extremely quick [10], while the nanometric dimension improves the interaction with the cells and the extracellular matrix [11]. Simultaneously, MBGNs can be doped with different trace elements which are constituents of several enzymes and proteins involved in biological functions [12]. Hence, the ions released from the structure of the MBGN in the body could provide several therapeutic effects such as antibacterial activity, angiogenesis, and osteogenesis [12,13,14].

One of these therapeutic cations is Zn^2+^, which upregulates the expression of different genes involved in angiogenesis and anti-inflammatory processes [15,16]. Zn^2+^ also stimulates the production of alkaline phosphatase (ALP) [17], while it assures an inhibitory effect on a wide spectrum of Gram-negative and Gram-positive bacteria [17,18,19]. It is believed that Zn^2+^ disrupts the intrabacterial metal homeostasis, which leads to an increase in the oxidative stress generated by the presence of the reactive oxygen species (ROS) [20,21]. On the other hand, Mg^2+^ is directly involved in osteogenesis through the activation of the Notch1 cellular pathway [22]; it also stimulates the overexpression of COL10A1 genes [23]. In vitro bioactivity tests carried out on Mg^2+^-doped glass ceramics have shown that the presence of Mg^2+^ leads to accelerated bioactive behaviour, while it stimulates the proliferation of osteoblasts and the formation of the new bone [24].

S53P4 (53% SiO_2_, 4% P_2_O_5_, 20% CaO, 23% Na_2_O -%wt), one of the currently used BG for medical applications [25], represents the starting point of this work. However, recent studies have shown that a high content of Na^+^ accumulated in the body is associated with an increase rate of stroke, heart failure and chronic kidney diseases [26,27]. Moreover, whereas the original S53P4 was synthesised by melt quenching, in the present study, a microemulsion assisted sol-gel method was used. In the initial composition, Na_2_O played a key role in shortening the melting interval and lowering the temperature of the precursor mixture [28]. Because the chosen synthesis method involved low processing temperatures [29], Na_2_O was no longer needed. Knowing that Mg^2+^ and Zn^2+^ stimulate different cellular signalling pathways involved in the process of bone tissue regeneration, Na_2_O was equimolecularly substituted with MgO and/or ZnO. Thus, the purpose of this study was to develop and characterise for the first time in the literature MBGNs containing four or five components in the SiO_2_-P_2_O_5_-CaO-MgO-ZnO compositional system. Additionally, until now, there were no studies showing that P_2_O_5_ can be incorporated into the silica network when the microemulsion assisted sol-gel method was used. Our study demonstrates that P_2_O_5_ can be included in the MBGN structure, while the presence of Zn^2+^ leads to a delay of the bioactivity process.

## 2. Materials and Methods

### 2.1. Materials

Hexadecyltrimethylammonium bromide (CTAB, ≥98%, Sigma-Aldrich, Missouri, MO, USA), ethyl acetate (EA, ≥99.5%, Riedel-de-Haën, Seelze, Germany), ammonia (NH_3_, ≥25%, S.C. Chimreactiv S.R.L., Bucharest, Romania), tetraethyl orthosilicate (TEOS, ≥99%, Sigma-Aldrich), calcium nitrate tetrahydrate (Ca(NO_3_)_2_⋅4H_2_O, ≥99%, Sigma-Aldrich), di-ammonium hydrogen phosphate ((NH_4_)_2_HPO_4_, ≥98%, Fluka Chemie AG, Buchs, Switzerland), magnesium nitrate hexahydrate (Mg(NO_3_)_2_⋅6H_2_O, 99%, Sigma-Aldrich) and zinc acetate dihydrate (Zn(CH_3_COO)_2_⋅2H_2_O, ≥98%, Sigma-Aldrich) were the analytical grade chemicals used for the synthesis of the MBGNs.

### 2.2. Synthesis of Doped MBGNs

Three types of MBGNs with the nominal compositions being presented in Table 1 were developed by a slightly modified microemulsion assisted sol-gel method in accordance with other similar studies [30].

Firstly, 2% (*w*/*v*) CTAB was solubilized in distilled water (DW) at 50 °C for 30 min. until the solution became clear. After switching off the temperature, EA was slowly added in a ratio CTAB:EA = 1:14 (*w*/*v*), followed by 30 min of stirring before dropwise adding NH_4_OH to achieve a pH of 8.5. The mixture was left under magnetic stirring for 30 min. and then TEOS was added in an amount 6 times greater than CTAB. During this time, the required quantity of (NH_4_)_2_HPO_4_ was dissolved in the minimum amount of DW. The obtained solution was slowly dripped to the previous emulsion while assuring that the pH did not go over 9.2. Then at every 40 min. Ca(NO_3_)_2_⋅4H_2_O, Mg(NO_3_)_2_⋅6H_2_O and/or Zn(CH_3_COO)_2_⋅2H_2_O were subsequently added. After 4 h of homogenisation, a white precipitate was formed and collected by centrifugation at 7000 rpm/5 min. Prior to drying the collected particles at 60 °C for 12 h, they were washed 3 times with DW. In the end, the powders were calcinated at 600 °C for 3 h with a heating rate of 2 °C per minute.

### 2.3. Physicochemical Characterisation of Mesoporous Bioactive Glass Nanoparticles

The X-Ray diffraction was performed with a Shimadzu XRD 6000 (Shimadzu, Kyoto, Japan) diffractometer equipped with a Cu Kα radiation tube (λ = 1.5406 Å). The data were collected in the 2θ range of 5°–65° at a scan speed of 2°/min and a dwell time of 0.6 s. The structure of the MBGNs was assessed by Fourier-transform infrared spectroscopy analysis (FT-IR, Thermo iN10-MX Fourier, Thermo Fischer Scientific, Waltham, MA, USA). The FT-IR spectra were acquired in absorbance mode between 4000 cm^−1^ and 400 cm^−1^ and a resolution of 4 cm^−1^. Nitrogen physisorption analysis was carried out to assess the porous nature of the particles. More precisely, the samples were degassed for 5 h at 150 °C before introducing them in a Micrometrics ASAP 2020 device (Micrometrics Instruments Corporation, Norcross, GA, USA). The specific surface together with the pore size distribution and the mean pore volume were determined by applying the Brunauer-Emmett–Teller (BET) and Barrett–Joyner–Halenda (BJH) methods. An Inspect F-50 high-resolution scanning electron microscope (SEM) coupled with an energy-dispersive X-ray spectroscopy (EDX) detector (Thermo Fisher—former FEI, Eindhoven, The Netherlands) was also used. Based on SEM micrographs, 200 particles of each composition and of each type were measured with ImageJ software 1.32j (NIH, Bethesda, MD, USA). The SEM analysis had the purpose of observing the morphologies of the particles, while information about the elemental composition of the samples immersed for 14 days in simulated body fluid (SBF) was obtained by the EDX. Transmission electron microscopy (TEM) was performed with a TecnaiTM G2 F30 S-TWIN high resolution electron microscope (FEI Company, Hillsboro, OR, USA). Before starting the analysis, a small amount of each powder was homogeneously dispersed in pure ethanol by 10 min of ultrasonication. Even though the images were recorded at different magnifications, all of them were taken at 300 kV in transmittance mode.

### 2.4. In Vitro Bioactivity in Simulated Body Fluid (SBF)

The ability of the MBGNs to form a layer of phosphates on their surface was investigated by in vitro bioactivity in SBF. For this analysis, the calcinated powders were uniaxially pressed at approximatively 2tnf in 13 mm diameter pellets, while the SBF was freshly prepared as previously described by Kokubo et al. [31]. Subsequently, the samples in triplicate were immersed in SBF at a concentration of 1 mg/mL. They were further kept in a laboratory water bath at 37 °C for 3 testing times: 3, 7, and 14 days, without changing or refreshing the SBF during this time. At every predetermined immersion time, both the pH and the conductivity of the SBF were measured and the obtained results are being shown as the mean value ± standard deviation. Before drying the pellets for 12 at 60 °C, they were gently washed with DW. In the end, the composition, and the morphology of the immersed MBGNs were determined by FT-IR and SEM/EDX.

### 2.5. Antibacterial Assay

*Escherichia coli* TOP10 was used to investigate the antibacterial activity of the MBGNs. *E. coli* suspension was prepared in LB Broth media (Miller) (L2542, Sigma-Aldrich), while the MBGNs pellets were sterilized by exposing to UV light each side of the sample for 20 min. Once the MBGNs were transferred to 24-well plates (TPP), each pallet was inoculated with 150 μL of *E. coli* suspension and 1.5 ml of sterile Phosphate Buffered Saline (PBS, P3813, Sigma-Aldrich). The control groups consisted of *E. coli* suspension in LB Broth media and sterile PBS. Afterwards, the samples were incubated at 37 °C for 20 h and 40 h in an atmosphere containing 5% CO_2_. A DS-11 FX+ Spectrophotometer (DeNovix, Wilmington, DE, USA) was used to determine the optical density (OD) of the *E. coli* at 600 nm [32].

### 2.6. In Vitro Cytocompatibility Assay

#### 2.6.1. GSH Assay

Because the presence of MBGNs might induce intracellular oxidative stress, GSH (glutathione) assay was performed. This assay is based on the ability of GSH to convert luciferin derivates to luciferin, reaction which is further coupled with Ultra-Glo Recombinant Luciferase. As a result of these subsequential conversions, a luminescent signal is generated, which is proportional to the level of GSH. GSH is directly involved in the neutralisation of the reactive oxygen species (ROS), thus, an increase of the GSH intracellular level corresponds to a higher oxidative stress.

For GSH-Glo Glutathione assay (GSH-GloTM, Promega, Madison, WI, USA), the THP-1 human leukaemia monocytic cells were seeded into 96-well plates at a density of 3000 cells/well in a supplemented DMEM medium. More precisely, besides DMEM, the growing media contained 10% fetal bovine serum and 1% streptomycin/neomycin, penicillin. The cells were incubated at 37 °C for 24 h before adding the MBGNs pellets. For the control group, the cells were grown in DMEM, 10% fetal bovine and 1% streptomycin/neomycin, penicillin. Afterwards, they were treated the same way as the groups exposed to MBGNs. After 72 h of contact, 100 µL of 1X GSH-Glo reagent was added in each well, followed by another 15 min of incubation at 37 °C. The generated luminescence was quantified with a Microplate Luminometer Centro LB 960 (Berthold, Bad Wildbad, Germany).

#### 2.6.2. MTT Assay

The (3-(4,5-dimethylthiazol-2-yl)-2,5-diphenyltetrazolium bromide) (MTT) assay was carried out to determine the influence of the MBGNs on the cell proliferation, and viability. In viable cells, nicotinamide adenine dinucleotide phosphate (NADPH)-dependent cellular oxidoreductase enzymes are directly involved in the mitochondrial metabolization process of the MTT. Hence, as a result of the enzymes conversion, the water-soluble yellow MTT is transformed into purple insoluble formazan. The obtained salt is then dissolved in isopropanol and the optical density of the solution is spectrophotometrically quantified. A higher value of the collected signal corresponds to an increase of the cell proliferation, signal which is directly proportional to the cell viability.

In this study Vybrant™ MTT Cell Viability Assay (Thermo Fisher Scientific, Waltham, MA, USA) together with the THP-1 human leukaemia monocytic cells were used for the MTT assay. Firstly, the sterilised MBGNs disks were place separately in each well of 96-well plates, followed by cell seeding at a density of 3000 cells/well. DMEM medium (Sigma-Aldrich, Missouri, MO, USA), 10% fetal bovine serum and 1% streptomycin antibiotics (Sigma–Aldrich, Missouri, MO, USA) were the components of the growing medium. For the control group, the THP-1 cells were grown in DMEM, 10% fetal bovine and 1% streptomycin/neomycin, penicillin. Afterwards, they were treated the same way as the groups exposed to MBGNs. While the cells were incubated at 37 °C in the presence or not of the MBGNs for 24 h and 72 h, the culture medium was changed twice a week to assure that the optimal growing conditions were fulfilled. Once the established testing time passed, 15 mL of MTT was added in each well. After another 4 h of incubation, 10 mL of a solution containing 1 mg of sodium dodecyl sulphate and 10 ml HCl (0.01 M) was subsequently added. When the formazan crystals became solubilised, TECAN Infinite M200 spectrophotometer (Männedorf, Switzerland) was used to quantify the optical density of the obtained solution at 570 nm. These results were further used together with Equation (1) to determine the relative cell viability.

Relative cell viability (%) = Sample (OD − 570 nm)/Control (OD − 570 nm) × 100

### 2.7. Statistical Analysis

The experimental results were calculated as the mean value ± standard deviation (SD) between the three measurements made for each sample. Additionally, Origin 2021 (OriginLab, Northampton, MA, USA) software with one-way ANOVA module and post hoc Turkey test was used to compare the mean differences. In the captions of each figure where this analysis was carried out, the intervals of confidence and the statistically significant values are presented in detail.

## 3. Results and Discussion

### 3.1. Physicochemical Characterisation

**Figure 1:**
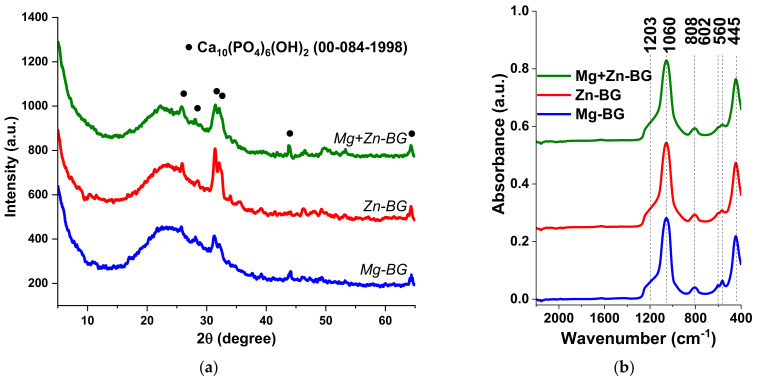

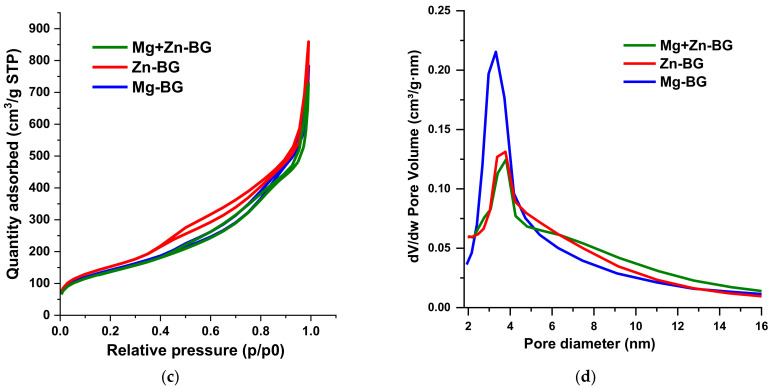
XRD patterns (**a**), FT-IR spectra (**b**), N_2_ physisorption isotherms (**c**) and BJH pore size distributions (**d**) of synthesised MBGNs.

The influence of different types of oxide modifiers on the structural characteristics of the MBGNs was studied by XRD, and in Figure 1a for all samples, a wide halo can be seen between 2θ = 15° and 35°, which shows the amorphous nature of the silicate BG. In addition, low-intensity peaks corresponding to HAp (ASTM 084-1998) could be identified, suggesting a poorly crystalline degree. Furthermore, HAp formed in the composition containing Zn^2+^ has a higher degree of crystallinity compared with those with MgO, which was in accordance with our previous findings [33]. Hence, it can be said that all the samples have a typical glass-ceramic structure, while the presence of ZnO led to an increase of the crystallinity of HAp.

To have a better understanding of the BG network, FT-IR analysis was carried out (Figure 1b). Specific Si-O-Si rocking vibration and Si-O-NBO asymmetric stretching bands could be identified around 445 cm^−1^ and 808 cm^−1^ and 1060 cm^−1^, respectively [34,35]. Moreover, two other bands distinguished at 560 cm^−1^ and 602 cm^−1^ are specific to O-P-O bending and stretching vibration of PO_4_^3−^ groups in HAp [36]. The broad shoulder centred at 1203 cm^−1^ corresponds to the signal coming from both Si-O-P stretching modes and Si-O-Si symmetric stretching vibration [35]. Therefore, for all samples, the FT-IR spectra proves the successful incorporation of the P_2_O_5_ in the silicate network, network which is partially disrupted due to the incorporation of different cations in this structure.

The textural proprieties of the MBGNs were investigated by N_2_ adsorption-desorption analysis. The obtained isotherms were further compared to the different type of theoretical models as stated by the International Union of Pure and Applied Chemistry (IUPAC). The identified type IV of isotherm reveals that all the particles were mesoporous (Figure 1c) [37]. Moreover, the isotherms had a H3 hysteresis loop, which means that slit-shape pores were present in the structure of the MBGNs [37]. Because the materials were mesoporous, the specific surface, total pore volume, and mean pore diameter (Table 2) were calculated according to BET and BJH methods.

Even though all MBGNs had high specific surfaces (>400 m^2^/g), there were slight variations between different compositions. All samples showed a monomodal BJH pore size distribution (Figure 1d), in the range of 2 to 15 nm, with no differences between them. The presence of such pores in the structure of the BG might have led to a higher reactivity and an bioactive behaviour, thus accelerating the integration of the biomaterial in the body. Additionally, it induced a higher specific surface, which means an increased interaction with the liquids present in the human body and with the cells. At the same time, drugs could be loaded in the mesopores, thus assuring a controlled drug delivery for longer periods of time compared with the conventional drug delivery systems.

**Figure 2:**
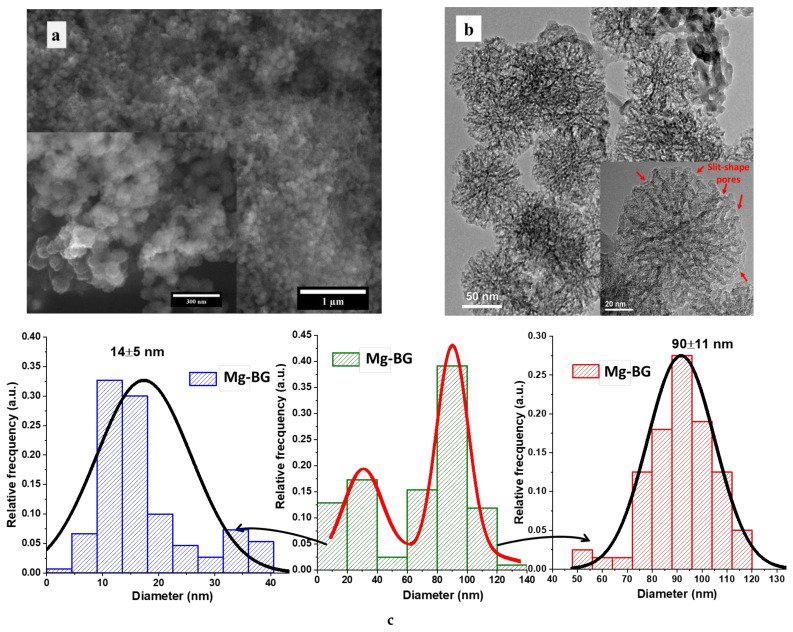

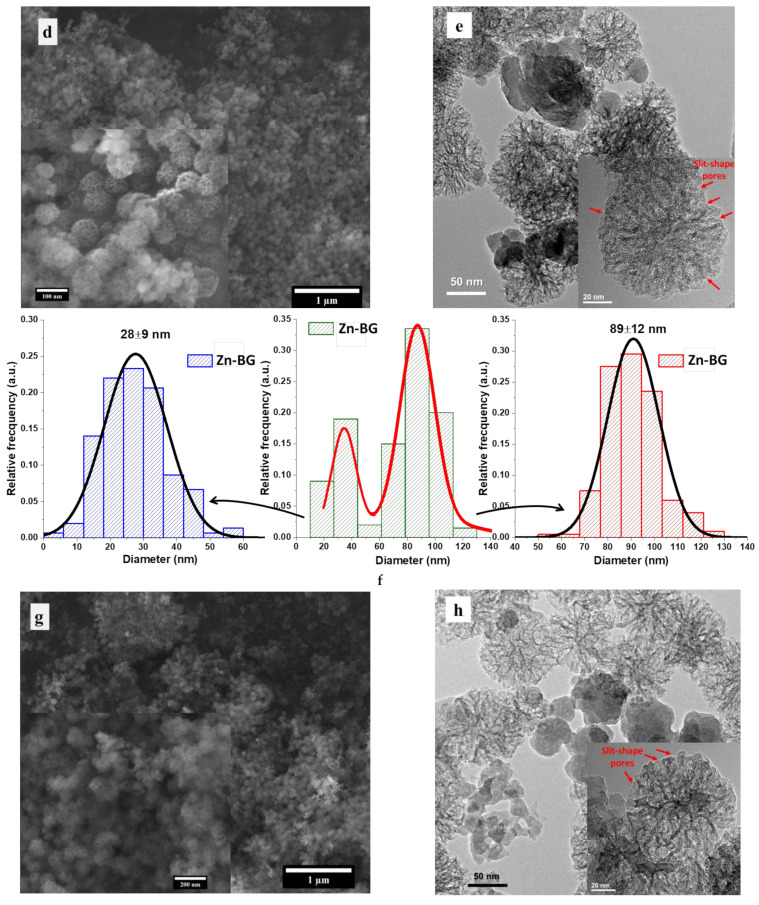

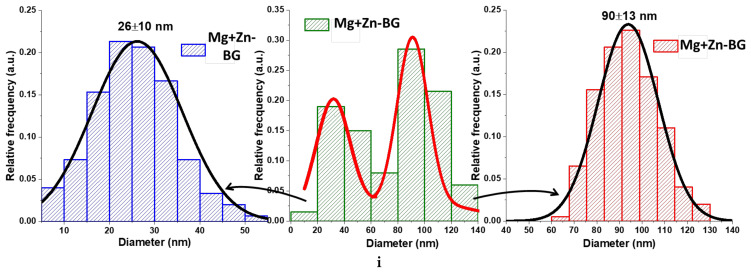
SEM and TEM images of Mg-BG (**a**,**b**), Zn-BG (**d**,**e**), Mg+Zn-BG (**g**,**h**) together with the particle size distribution and mean diameter for Mg-BG (**c**), Zn-BG (**f**), Mg+Zn-BG (**i**).

The SEM and TEM analysis (Figure 2a,b,d,e,g,h) not only confirm the porous nature of the MBGNs, but it also depicts their morphology. Thus, for each composition, MBGNs can be divided in two different families as revealed by the bimodal particle size distributions (Figure 2c,f,i). MBGNs with a quasi-spherical shape and large mesopores (MBGNs-b) on the surface seem to be in a higher amount compared to the other type of MBGNs. The second family has a smaller particle size and a smoother surface together with an elongated morphology (MBGNs-s). Moreover, if the MBGNs-b have an interconnected network of radial slit-shape pores, the other class of particles are more compact. Despite the highlighted dissimilarities between MBGNs, all of them have a monomodal particle size distribution, with the average diameter being in the range characteristic to nanoparticles. Even though the concentration and the type of oxide modifiers were changed between samples, the morphology, the structure, and the shape of MBGNs-b were not affected. However, the presence of ZnO led to an increase of the diameter of the MBGNs-s. The highly dispersity and homogeneity of all MBGNs along with the low tendency to form agglomerates are specific to the synthesis method used to obtain the particles.

### 3.2. In Vitro Bioactivity in Simulated Body Fluid (SBF)

A key factor involved in the acceptance process of the BG in the human body as a synthetic bone graft is its ability to form a uniform layer of calcium phosphate on the surface. To prove this propriety, in vitro bioactivity in SBF was performed.

**Figure 3:**
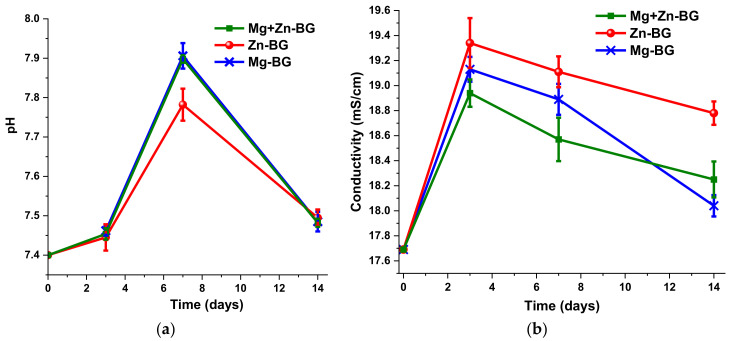
pH (**a**) and conductivity (**b**) of the SBF containing MBNGNs after 0, 3, 7 and 14 days of immersion.

While the MBGNs were immersed in SBF, the pH and the conductivity of the liquid were measured. As it can be seen from Figure 3, during the first 3 days of soaking both the pH and the conductivity rise. However, between the 3rd and the 7th day, the pH increased with greater velocity than in the previous testing interval, whereas the conductivity began to decrease. This might suggest that even though on the surface of the MBGNs a precipitation process took place, ions are still released from the material. After the 7th day of testing, a precipitate might have been formed, as the pH and the conductivity decreased. Despite the fact that all the samples had a similar behaviour, there were significant differences between the maximum pH values reached after 7 days of testing. The lower value corresponds to the composition with ZnO, most probably due to the lower basicity of this oxide compared with MgO or because it has the highest degree of crystallisation, which delays the ion release.

To confirm these observations and to investigate the morphology and the nature of the newly formed phase, SEM and XRD analysis were carried out.

**Figure 4:**
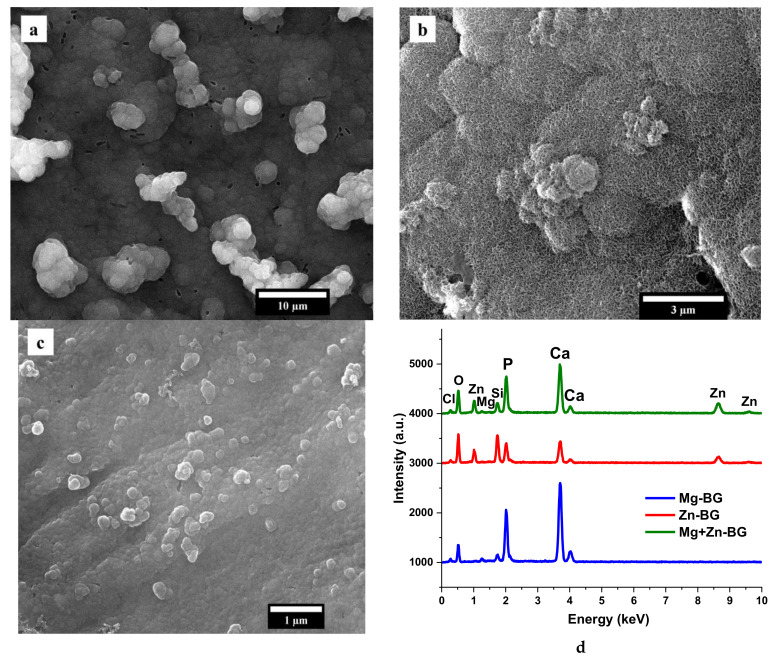
Scanning electron microscopy (SEM) micrographs of Mg-BG (**a**), Zn-BG (**b**) and Mg+Zn-BG (**c**), together with the EDX spectra of MBGNs (**d**) after 14 days of immersion in SBF.

SEM images together with the EDX spectra (Figure 4) show that the surfaces of all the samples are entirely covered with a uniform layer of calcium phosphates (CaP). Although the precipitates identified on Mg-BG and Mg+Zn-BG have a similar cauliflower morphology, the presence of ZnO led to a smaller dimension of these agglomerates. In contrast with the Mg^2+^-doped MBGNs, for the composition containing only ZnO the calcium phosphate layer has a needle like shape. Moreover, as shown in the EDX spectra, Mg^2+^ might favour the formation of the CaP layer on surface of the samples. This can be explained by the fact that the intensity of interference corresponding to Si from Mg-BG sample is smaller than in Zn-BG and Mg+Zn-BG samples, and the intensity of interferences corresponding to Ca and P are higher in Mg-BG sample than in the other two samples.

**Figure 5:**
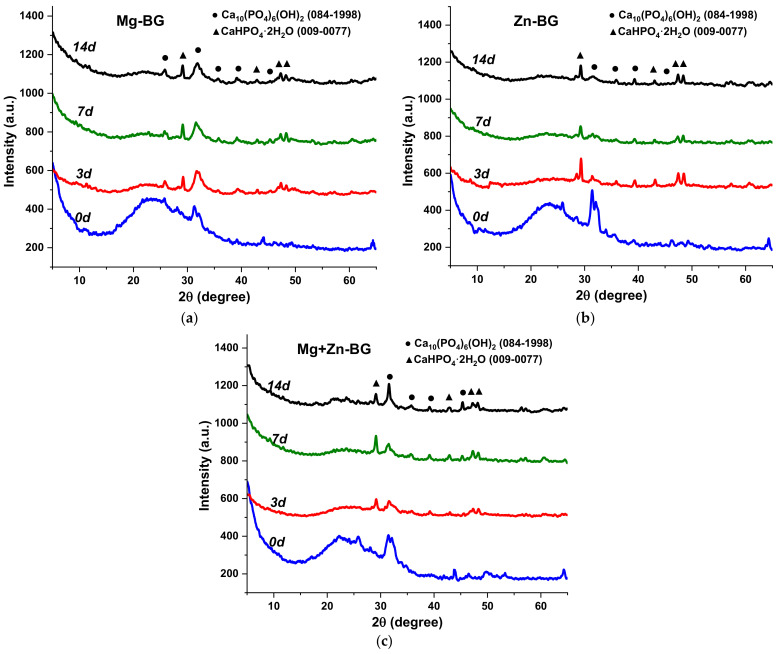
XRD patterns (**a**–**c**), of MBGNs after being soaked in SBF for 0, 3, 7 and 14 days.

The nature of CaP observed in the SEM micrographs was assessed by XRD (Figure 5), and on the 3rd day of immersion, the intensity of the broad band between 2θ = 5° and 35° decreased. This clearly proves that the phosphosilicate network starts dissolving as a result of the interaction between MBGNs and SBF. At the same time, a new phosphate phase, brushite (dicalcium phosphate dihydrate-DCPD; CaHPO_4_⋅2H_2_O; ASTM 009-0077), could be identified. When the testing time was increased to 7 days, a higher degree of crystallinity was achieved for brushite, while the intensity of the peaks characteristic to HAp did not rise significantly. As brushite is known to have low stability in basic environment [37,38], it is not surprising that there was an important decrease in its crystallinity on the 14th day. It could be said that brushite dissolves and most probably reprecipitates as poorly crystalline HAp. Even though these observations are generally valid for all the samples, there are slight changes when it comes to the degree of crystallinity of the newly formed phase. ZnO slowed down the formation of both brushite and HAp, while the conversion of brushite was not affected. These results are in accordance with the SEM observations regarding the thickness of the CaP layer.

Hence, it can be concluded that no matter the composition, the obtained MBGNs have a bioactive behaviour in SBF. The nanometric dimension of the particles and the high specific surfaces leads to an increased dissolution rate compared with traditional bioactive glasses. However, compared with MgO, ZnO delayed the formation of these CaP with an important change of the morphology of the CaP. When equal amounts of MgO and ZnO were used, the cauliflower shape of the CaP is common to the composition containing only MgO, whereas ZnO decreased the crystallinity of the CaP layer. In previous studies, the same effect of ZnO on the mineralisation process of the BG was highlighted [33,39,40].

Neščáková et al. [39] previously reported the synthesis of Zn-doped MBGNs in the CaO-SiO_2_ system. The same binary system was also used in another study, where the effect of different amounts of MgO was investigated. In comparison with their results, in this study, the compositional system was more complex, and the obtained particles had superior textural proprieties and smaller diameters. Additionally, for Zn^2+^-MBGNs, Neščáková et al. proved that no matter the testing time, even a small content of ZnO delayed the precipitation of CaP. Because the MBGNs were designed for synthetic bone grafts, the material should be able to directly bond to the bone. Thus, we showed that even when the amount of ZnO was high, the material immersed in SBF was still covered by a layer of CaP.

Huang et al. [41] studied the structural and morphological changes induced by various proportions of P_2_O_5_. The synthesised particles were not in the nanometric domain, and the analysis required showing the mesoporous structure was not carried out. The results of the present study are even more important because there are only a few articles where P_2_O_5_ was successfully incorporated in the glass structure using the microemulsion-assisted sol-gel method. In previous experiments, triethyl phosphate (TEP) was used as a P_2_O_5_ source. TEP hydrolysis take place more slowly for TEP than for TEOS, which means that the silica network is already formed when TEP could enter in the glass network. Moreover, TEP is highly soluble in DW, and it might be removed from the mixture during the washing steps [42]. However, in our study we have used a different P_2_O_5_ precursor, thus assuring that P_2_O_5_ was present in the glass structure.

### 3.3. Antibacterial Activity

**Figure 6:**
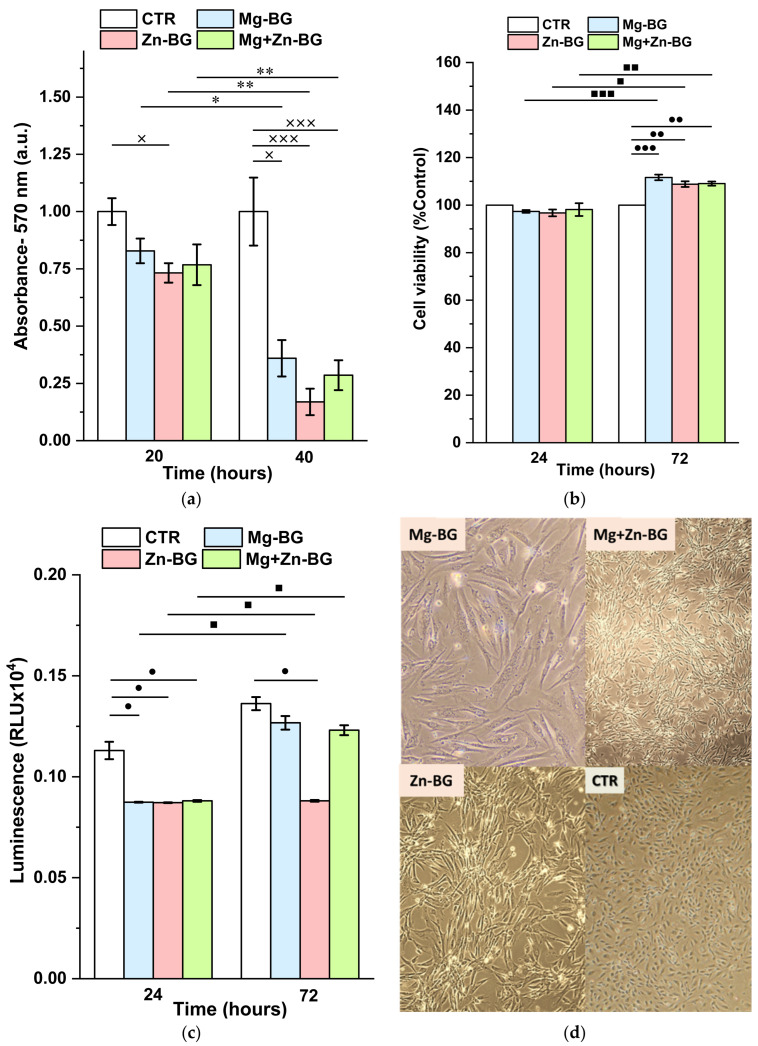
Antibacterial assay of the MBGNs on *E. coli* strain at different testing times (**a**), Results of the GSH assay on THP-1 cells after being cultured on the MBGNs for 24 h and 72 h (**b**); THP-1 cell viability after 24 h and 72 h of contact with the MBGNs (**c**); Light microscopy images of the THP-1 cells cultured for 72 h on MBGNs disks (**d**) (samples prepared in triplicate; for the antibacterial assay - × *p* < 0.05, ××× *p* < 0.001 significant difference between the CTR and MBGNs, * *p* < 0.05 * *p* < 0.01 significant differences between the same composition at different testing times; for the cytocompatibility test - • *p* < 0.05, •• *p* < 0.01, ••• *p* < 0.001 significant differences between CTR and Mg-BG/ Zn-BG/ Mg+Zn-BG; • *p* < 0.05; ▄ *p* < 0.05, ▄ ▄ *p* < 0.01; ▄ ▄ ▄ *p* < 0.001 significant differences between the same compositions at 24 h and 72 h).

Within the first 20 h of testing, there were no significant differences between the control and Mg-BG or Mg+Zn-BG (Figure 6a), although for Zn-BG, an inhibitory effect against *E. coli* strain was recorded. This might be due to the known antibacterial effect of the Zn^2+^ ions that are released from the structure of the MBGNs. When the samples were evaluated after 40 h, for all compositions, the bacterial viability decreased compared with the control and the corresponding values at 20 h. Possible explanations for this phenomenon might be the high pH or as the accumulation of a high amount of the released ions.

### 3.4. In Vitro Biocompatibility Assay

Because the MBGNs were designed to be implanted in the body, it was of utmost importance to show that they were not cytotoxic. Thus, GSH and MTT assays were carried out.

The GSH results (Figure 6c) reveal that after 24 h of contact, a significant reduction in the level of oxidative stress was recorded for all the samples in comparison with the control. At 72 h post incubation, only the presence of Zn-BG reduced the number of free radicals compared to the control. Even though for all samples, the intracellular glutathione values were higher at 72 h than at 24 h, this increase was not statistically significant in comparison with the control.

Not only did the MBGNs not induce intracellular stress, they also did not affect the cell proliferation. The quantitative MTT results show that in the first 24 h, the cell viability of the THP-1 cells was not affected by the presence of the MBGNs disks. When the cells were evaluated at 72 h, for all MBGNs, a higher cell viability was achieved in comparison with the control and the 24 h values. It should be mentioned that no significant differences could be distinguished among different MBGN compositions. From a qualitative point of view, the presence of the inorganic material led to a change in the cell morphologies. If the cells grown on the control have a spherical shape characteristic to monocytes, those evaluated after being in contact with the MBGNs started to spread on the surface. Thus, the cells adopt an elongated shape with a clear emission of cytoplasmatic pseudopods.

In the end, it could be said that the MBGNs did not determine an increase of the intracellular oxidative level, while the cell viability was slightly enhanced, especially on the short testing times.

## 4. Conclusions and Further Perspectives

In this present work, the SiO_2_-P_2_O_5_-CaO-MgO-ZnO compositional system was used to successful synthesise MBGNs through microemulsion-assisted sol-gel method. The obtained particles had a typical structure of phosphosilicate BG with a poorly crystalline phase identified as HAp. Among different compositions, the presence of ZnO led to an increase of the crystallinity of HAp. The N_2_ physisorption analysis proved that all the particles were mesoporous with slit-shaped pores. However, a slightly decrease in the specific surface and a higher porosity were achieved for the composition containing Zn^2+^, while the opposite was recorded for the MBGNs with equal amounts of MgO and ZnO. These results were in accordance with the SEM images where large pores could be seen on the surface of the particles. Moreover, together with TEM micrographs, two families of highly dispersed, homogenous and nanometric MBGNs could be highlighted. If the first type of MBGNs had a spherical morphology with an interconnected network of mesopores, the second family was characterized by a smaller dimension and a more compact internal structure. Even though all the big MBGNs had similar diameters no matter the composition, the presence of ZnO determined a slightly increase of the small particles. In vitro bioactivity tests revealed that the surfaces of all MBGNs were covered by a uniform layer of brushite which slowly dissolves and reprecipitates most probably as highly substituted Hap. All MBGNs exhibited an antibacterial effect, while the cell viability and the oxidative stress were not affected. In the future, specific cellular tests should be carried out to exactly understand how these ions activate different cellular behaviours. Additionally, MBGNs could be further used for drug loading and delivery or as inorganic fillers for bone tissue engineering scaffolds.

## Figures and Tables

**Table 1:** Nominal compositions of MBGNs (%wt).

Symbol	SiO_2_	P_2_O_5_	CaO	MgO	ZnO
Mg-BG	53	4	20	23	
Zn-BG	53	4	20	-	23
Mg+Zn-BG	53	4	20	11.5	11.5

**Table 2:** Results of the N_2_ physisorption analysis carried out for the MBGNs.

Symbol	Specific Surface (m^2^/g)	Average Pore Diameter (nm)	Total Pore Volume (cm^3^/g)
Mg-BG	482	8.0	1.35
Zn-BG	476	8.7	1.55
Mg+Zn-BG	509	8.1	1.24

## Data Availability

Not applicable.
